# Development and Validation of a Patient-Reported Outcome Questionnaire for Children Undergoing Removable Orthodontic Treatment

**DOI:** 10.3390/dj14090543

**Published:** 2026-09-01

**Authors:** Panaite Tinela, Carmen Diana Nicoleta Savin, Ana Sîrghie, Teona Ana-Maria Tudorici, Irina Bamboi, Anca Rapis, Ana Nemțoi, Ciprian-Iulian Axinte, Carina Ana-Maria Balcos

**Affiliations:** Grigore T. Popa University of Medicine and Pharmacy Iasi, 16 Universitatii Str., 700115 Iasi, Romania; tinela-panaite@umfiasi.ro (P.T.); carmen.savin@umfiasi.ro (C.D.N.S.); ana.petcu@umfiasi.ro (A.S.); teona.tudorici@umfiasi.ro (T.A.-M.T.); irina.bamboi@umfiasi.ro (I.B.); anca.stupu@umfiasi.ro (A.R.); ana.nemtoi@umfiasi.ro (A.N.); carina.balcos@umfiasi.ro (C.A.-M.B.)

**Keywords:** compliance, orthodontic treatment, questionnaire development, psychometric validation, psychosocial impact, removable appliances, survey

## Abstract

**Background/Objectives**: Patient-reported outcome measures (PROMs) complement clinical indicators in orthodontics, yet validated instruments for children undergoing removable orthodontic treatment remain limited. This study aimed to develop and validate a questionnaire assessing compliance and physical discomfort, psychosocial impact, and perceived support. **Methods**: A cross-sectional validation study included 250 children and adolescents (<14 years) treated with removable orthodontic appliances, regardless of treatment duration. The 14-item questionnaire was evaluated for internal consistency (Cronbach’s alpha), reliability (intraclass correlation coefficient, ICC), item discrimination (ANOVA), and construct validity using exploratory factor analysis with Varimax rotation. Sampling adequacy was assessed using the Kaiser–Meyer–Olkin (KMO) measure and Bartlett’s test of sphericity. **Results**: Internal consistency was satisfactory (Cronbach’s alpha: 0.747–0.823), with corrected item–total correlations above 0.30. ICC values indicated moderate reliability for single measures and good reliability for average measures. The KMO value was 0.713, and Bartlett’s test was significant (*p* < 0.001). Three factors explained 58.69% of the total variance, representing psychosocial impact, compliance/discomfort, and perceived support. **Conclusions**: The questionnaire demonstrated satisfactory reliability and construct validity for assessing patient-reported outcomes in children undergoing removable orthodontic treatment. It provides a practical instrument for evaluating compliance and physical discomfort, psychosocial impact, and perceived support in both clinical practice and research.

## 1. Introduction

Orthodontic treatment with removable appliances extends beyond the correction of malocclusion, as pain, discomfort, speech difficulties, and functional limitations may influence children’s physical comfort, daily functioning, psychosocial well-being, oral health-related quality of life, and overall treatment experience [1,2,3,4,5,6,7,8]. Speech-related difficulties are particularly relevant in pediatric orthodontic populations: a recent scoping review found that malocclusion during the deciduous and mixed dentition stages is frequently associated with speech sound disorders, with traits such as anterior open bite consistently linked to altered articulation and phonetic clarity in children, underscoring the need to capture speech-related functional impact when evaluating removable appliance treatment in this age group [9].

Patient-reported experiences may directly influence treatment adherence and clinical outcomes, and their systematic assessment has become an increasingly important component of patient-centered orthodontic care [10,11,12,13,14,15,16,17,18].

Successful treatment with removable orthodontic appliances depends largely on patient adherence, as consistent daily wear and active cooperation are essential for achieving optimal clinical outcomes, while pain, discomfort, speech difficulties, functional limitations, and psychosocial factors—including patient motivation, self-efficacy, parental support, appliance acceptance, and clinician–patient communication—may significantly influence compliance and, ultimately, treatment success [19,20,21,22,23,24,25,26,27,28].

Despite the availability of several patient-reported outcome measures (PROMs), important limitations remain. Many existing instruments were originally developed for adults or have not been fully validated for pediatric populations, raising concerns about their suitability for children undergoing orthodontic treatment [25,27]. In addition, widely used questionnaires are generally designed to assess broad aspects of oral health-related quality of life rather than issues specific to removable orthodontic treatment, such as treatment adherence, appliance-related discomfort, psychosocial impact, and perceived support [8].

Furthermore, orthodontic patients have reported that some existing questionnaires contain items that are difficult to understand or insufficiently relevant to their treatment experience, potentially compromising content validity [8]. These limitations highlight the need for a condition-specific, psychometrically validated instrument capable of comprehensively evaluating the multidimensional experience of children receiving removable orthodontic treatment.

Given the increasing emphasis on patient-centered orthodontic care, there is a growing need for condition-specific, psychometrically sound patient-reported outcome measures capable of comprehensively assessing the experiences of children undergoing removable orthodontic treatment [7,12]. Current recommendations emphasize that PROM development should be based on a robust conceptual framework, involve patients and clinical experts during item generation, and undergo comprehensive psychometric evaluation according to the COSMIN guidelines [17].

The aim of this study was to describe the systematic development and psychometric validation of a questionnaire designed to assess compliance, physical discomfort, and the psychosocial impact of removable orthodontic appliance treatment. The study outlines the stages of questionnaire development, content validation, pilot testing, and psychometric evaluation, providing a framework for the standardized assessment of patient-reported outcomes in orthodontic care.

More specifically, the study aimed to: (1) describe the sociodemographic and treatment-related characteristics of the participants; (2) assess daily compliance with appliance wear; (3) evaluate physical adaptation and discomfort associated with appliance use; (4) assess the functional impact of the appliance on speech; (5) explore psychological and social perceptions related to orthodontic treatment; (6) assess patient motivation and attitudes toward treatment; (7) evaluate the quality of doctor–patient communication; (8) explore the role of family support; and (9) assess the perceived need for additional motivational or psychological support.

## 2. Materials and Methods

### 2.1. Study Design

The study was designed as an observational, cross-sectional study aimed at evaluating the psychometric properties of a questionnaire developed to measure the experience of patients wearing removable orthodontic appliances. The cross-sectional design allowed for data collection at a single time point and was appropriate for assessing the reliability and construct validity of the instrument.

### 2.2. Participants and Selection Criteria

The study sample consisted of 250 participants—children and adolescents undergoing orthodontic treatment with removable appliances. Participants were selected based on the following inclusion criteria:

- Age under 14 years;
- Use of a removable orthodontic appliance regardless of duration of use;
- Ability to understand and complete the questionnaire, with parental assistance when necessary;
- Informed consent obtained from parents or legal guardians.

Patients with cognitive disabilities that could affect comprehension of the questionnaire items or the accuracy of responses were excluded. Participation was voluntary, and data were collected anonymously, in accordance with ethical principles of medical research.

Sample size adequacy for the planned psychometric validation procedures was considered during study design. For exploratory factor analysis, commonly cited recommendations suggest a minimum participant-to-item ratio of approximately 5–10:1, or an absolute sample size of at least 200–300 respondents, to obtain stable and generalizable factor solutions. With 250 participants and a 14-item questionnaire, the present study achieved a ratio of approximately 18 respondents per item, exceeding these thresholds.

### 2.3. Research Instrument

The instrument used was an original, structured questionnaire entitled “Experience of Removable Orthodontic Appliance Use” (File S1), developed to assess wear compliance, physical discomfort, and psychological and social impact of treatment, as well as the patient–orthodontist relationship and perceived support. The questionnaire comprises 14 items organized into four sections:

- Section I—General information (age, sex, duration of appliance use);
- Section II—Compliance and physical discomfort (5 items);
- Section III—Psychological and social perception (6 items);
- Section IV—Doctor–patient relationship and perceived support (3 items).

### 2.4. Data Collection Procedure

The questionnaires were administered in an online format during orthodontic consultations, under the supervision of medical staff. Participants were informed about the purpose of the study, the anonymous nature of their responses, and the exclusive academic use of the collected data. The study was conducted between July 2025 and December 2025. A total of 280 patients were invited to participate in the study. Of these, 250 agreed to participate and completed the questionnaire, while 30 declined participation. The response rate was therefore 89.3%.

### 2.5. Statistical Analysis and Psychometric Validation

The collected data were analyzed using the SPSS 26.0 (IBM Corp., Armonk, NY, USA). Psychometric validation of the questionnaire included the following steps:

- Internal consistency analysis, performed by calculating Cronbach’s alpha coefficients for each questionnaire section, along with item–total correlation analysis;
- Assessment of stability and agreement, using the intraclass correlation coefficient (ICC) for individual and average measurements;
- Analysis of variance (ANOVA) to examine the discriminative capacity of the items within each section;
- Exploratory factor analysis (EFA) using Principal Component Analysis (PCA) to assess construct validity;
- Evaluation of data suitability for factor analysis using the Kaiser–Meyer–Olkin (KMO) measure and Bartlett’s test of sphericity;
- Determination of the optimal number of factors based on Kaiser’s criterion (eigenvalues > 1) and inspection of the scree plot;
- Application of Varimax rotation with Kaiser normalization to obtain a clear and interpretable factor structure.

A statistical significance threshold of *p* < 0.05 was considered for all analyses.

## 3. Results

### 3.1. Section I—General Characteristics

The analysis of the age group distribution indicates that the majority of participants included in the study fell within the 10–13-year age range, accounting for 77.6% of the total sample (n = 194). The remaining 22.4% of participants (n = 56) belonged to the under-10 age group (Table 1). The cumulative distribution shows that the entire sample was covered up to the age of 13 years, confirming that the study population consisted predominantly of children and preadolescents.

The analysis of the sample distribution by sex revealed a predominance of female participants, who accounted for 57.6% of the study sample (n = 144). Male participants represented 42.4% of the sample (n = 106) (Table 1).

The analysis of the duration of removable orthodontic appliance use showed that the largest proportion of participants, 36.8% (n = 92), had been wearing the appliance for 6–12 months. A further 28.0% of respondents (n = 70) reported a duration of use exceeding one year. Shorter durations were reported by 22.8% of participants (n = 57), who had been wearing the appliance for 3–6 months, and by 12.4% (n = 31), who were within the first three months of treatment (Table 1). The cumulative distribution shows that 72.0% of respondents had been wearing the appliance for at least six months.

### 3.2. Section II—Compliance and Physical Discomfort

The reliability of Section II, assessing compliance with removable orthodontic appliance wear and the associated physical discomfort, was evaluated using Cronbach’s alpha. The five-item scale demonstrated good internal consistency (Cronbach’s α = 0.788; standardized α = 0.792), exceeding the recommended threshold of 0.70.

Mean item scores showed moderate variability (Table 2). The highest mean score was observed for “The removable appliance affects my ability to speak normally” (M = 3.80, SD = 1.13), followed by discomfort limited to the beginning of treatment (M = 3.57, SD = 1.11) and pain or physical discomfort during appliance wear (M = 3.48, SD = 1.30). Lower mean scores were found for compliance with the orthodontist’s recommendations (M = 3.01, SD = 1.01) and ease of adaptation to appliance wear (M = 3.12, SD = 1.24).

Cronbach’s alpha values if an item was deleted ranged between 0.727 and 0.779 and did not exceed the overall alpha coefficient of the scale, indicating that removal of any item would not result in a significant improvement in reliability (Table 2). Consequently, all items were retained in the final version of the scale. Additionally, the squared multiple correlation values supported the ability of the items to be explained by the overall scale structure, confirming the internal coherence of this section.

ANOVA demonstrated significant differences among the items of Section II—Compliance and Physical Discomfort (F = 34.708, *p* < 0.001; Table 3). The residual mean square was 0.777, and the overall mean score of the scale was 3.397. Variance between participants indicated individual differences in responses.

The ICC analysis demonstrated moderate agreement for single measures (ICC = 0.426, 95% CI: 0.366–0.488) and good reliability for average measures (ICC = 0.788, 95% CI: 0.743–0.826) (Table 4). The associated F test was statistically significant (F = 4.706, *p* < 0.001).

### 3.3. Section III—Psychological and Social Perception

The reliability of Section III, assessing the psychological and social perception of removable orthodontic appliance use, was evaluated using Cronbach’s alpha. The six-item scale demonstrated good internal consistency (Cronbach’s α = 0.823; standardized α = 0.828).

Mean item scores showed variability in participants’ psychological and social perceptions (Table 5). The highest mean values were recorded for increased confidence in one’s smile (M = 3.34, SD = 0.94) and the overall impact of the appliance on daily life (M = 3.31, SD = 0.95). Moderate mean scores were observed for experiences of ridicule or negative comments (M = 3.28, SD = 1.13) and the perceived negative impact of orthodontic treatment on emotional well-being (M = 3.19, SD = 1.09). The lowest mean scores were found for social embarrassment (M = 2.88, SD = 1.30) and motivation to continue treatment (M = 2.96, SD = 1.11).

Item–total statistics showed corrected item–total correlations ranging from 0.465 to 0.732, all above the recommended threshold of 0.30 (Table 5). Cronbach’s alpha if an item was deleted ranged from 0.770 to 0.818 and remained below the overall coefficient (α = 0.823), indicating that no item deletion would improve the internal consistency of the scale. Accordingly, all six items were retained.

ANOVA demonstrated significant differences among the items of Section III—Psychological and Social Perception (F = 14.062, *p* < 0.001; Table 6). The residual mean square was 0.670, and the overall mean score of the scale was 3.159. Variance between participants indicated individual differences in responses.

The intraclass correlation coefficient (ICC) demonstrated moderate agreement for single measures (ICC = 0.437; 95% CI: 0.381–0.496) and good reliability for average measures (ICC = 0.823; 95% CI: 0.787–0.855) (Table 7). The associated F test was statistically significant (F = 5.649, *p* < 0.001).

### 3.4. Section IV—Doctor–Patient Relationship and Perceived Support

The reliability of Section IV, assessing the doctor–patient relationship and perceived support, was evaluated using Cronbach’s alpha. The three-item scale demonstrated acceptable internal consistency, with a Cronbach’s alpha of 0.747 (0.752 for standardized items), supporting the reliability of this section.

The highest mean score was recorded for the item “The orthodontist provided clear explanations regarding how to wear and care for the removable appliance” (M = 3.50, SD = 0.78), followed by “I feel supported by my parents during orthodontic treatment” (M = 3.47, SD = 0.68) (Table 8). The lowest mean score was observed for the statement “I would benefit from more motivational or psychological support from the orthodontist” (M = 3.36, SD = 0.77). Overall, standard deviations were low, indicating limited variability in participants’ responses. Corrected item–total correlations ranged from 0.536 to 0.660, while Cronbach’s α if an item was deleted ranged from 0.575 to 0.709, indicating that removal of any item would not improve the internal consistency of the scale.

Analysis of variance (ANOVA) revealed statistically significant differences among the items of Section IV—Doctor–Patient Relationship and Perceived Support (F = 5.193, *p* = 0.006; Table 9). The residual mean square was 0.281, and the overall (grand) mean score of the scale was 3.443. Variance between participants indicated individual differences in perceived doctor–patient relationship and support.

The ICC analysis demonstrated moderate agreement for single measures (ICC = 0.496, 95% CI: 0.423–0.567) and good reliability for average measures (ICC = 0.747, 95% CI: 0.688–0.797) (Table 10). The associated F test was statistically significant (F = 3.955, *p* < 0.001).

### 3.5. Exploratory Factor Analysis (EFA)

The Kaiser–Meyer–Olkin (KMO) measure of sampling adequacy was 0.713, indicating that the data were suitable for exploratory factor analysis (Table 11). Bartlett’s test of sphericity was statistically significant (χ^2^ = 1303.255, df = 91, *p* < 0.001), supporting the factorability of the correlation matrix.

Communality values obtained from PCA ranged from 0.399 to 0.735, indicating adequate representation of the questionnaire items by the extracted factors (Table 12). The highest communalities were observed for the items assessing the emotional impact of orthodontic treatment (0.735), parental support (0.732), increased confidence in one’s smile (0.714), and the clarity of the orthodontist’s explanations (0.705), whereas the lowest values were recorded for speech impairment (0.399) and the perceived impact of the appliance on daily life (0.413).

PCA identified three components with eigenvalues greater than 1 (Table 13). These components explained 24.13%, 20.22%, and 14.34% of the total variance, respectively, accounting for 58.69% of the cumulative variance. After Varimax rotation, the explained variance was redistributed across the three components (23.25%, 19.79%, and 15.65%), with no change in the cumulative explained variance.

### 3.6. Scree Plot Analysis

**Figure 1:**
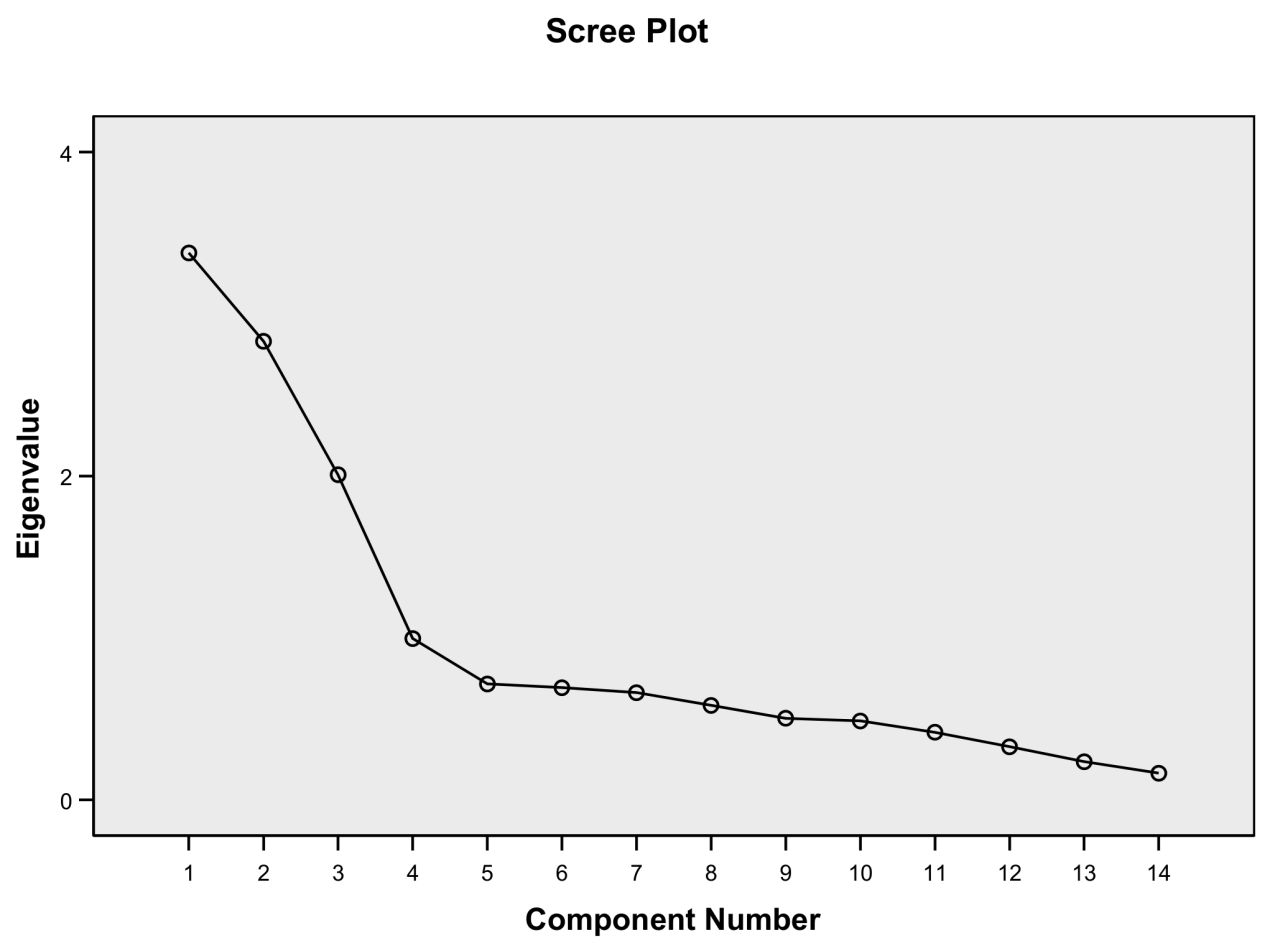
Scree plot of eigenvalues for exploratory factor analysis.

The scree plot showed a distinct inflection point after the third component, supporting the retention of three principal components (Figure 1). The first three components had eigenvalues greater than 1, whereas subsequent components contributed minimally to the total explained variance, consistent with Kaiser’s criterion.

### 3.7. Unrotated Component Matrix

**Figure 2:**
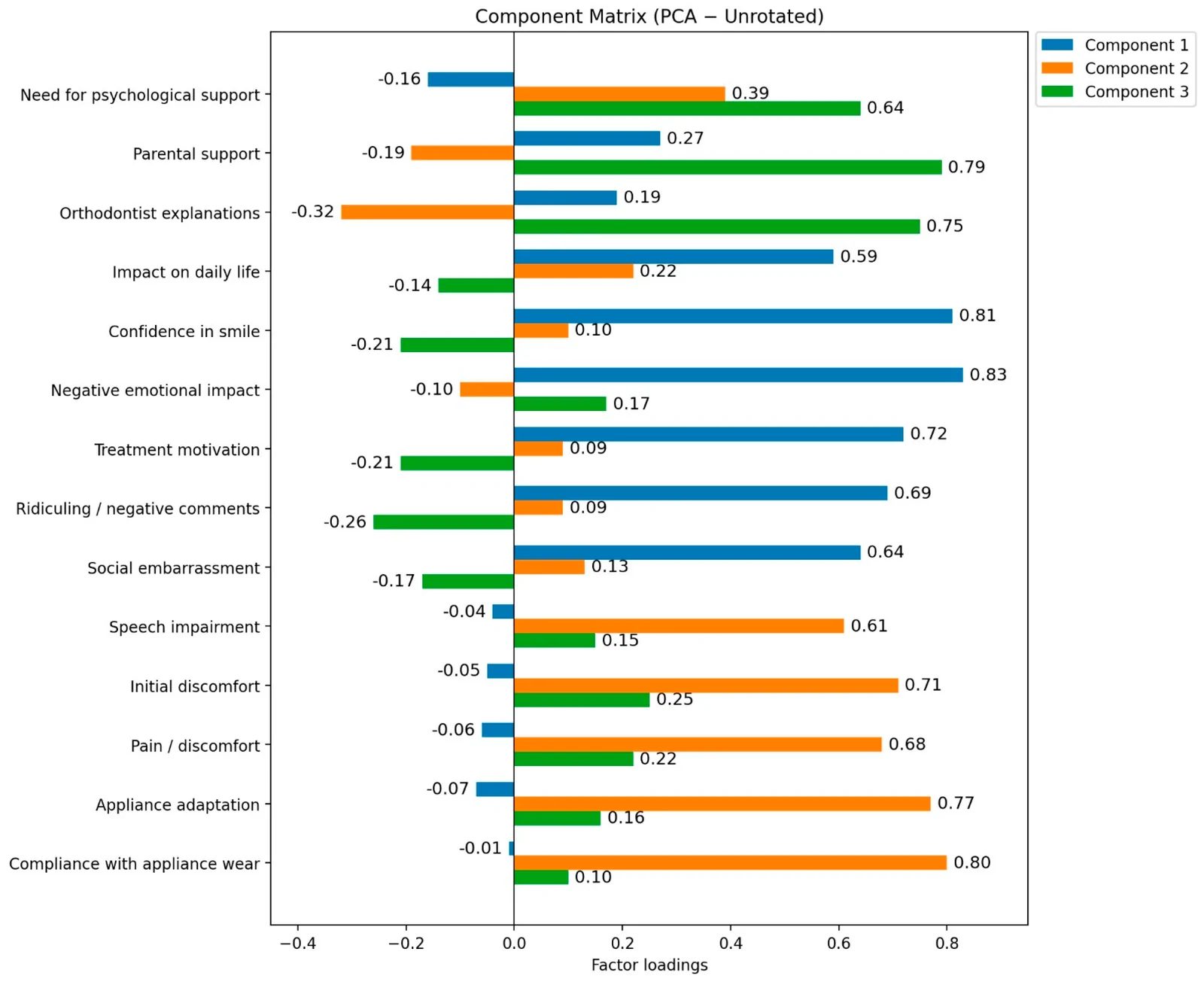
Graphical representation of the unrotated component matrix (PCA—no rotation).

The unrotated component matrix identified three components corresponding to the psychological and social impact of treatment, compliance and physical discomfort, and doctor–patient relationship and perceived support (Table 14; Figure 2). Factor loadings ranged from 0.587 to 0.801, with several items showing cross-loadings prior to Varimax rotation.

### 3.8. Varimax Rotation and Interpretation of Components

**Figure 3:**
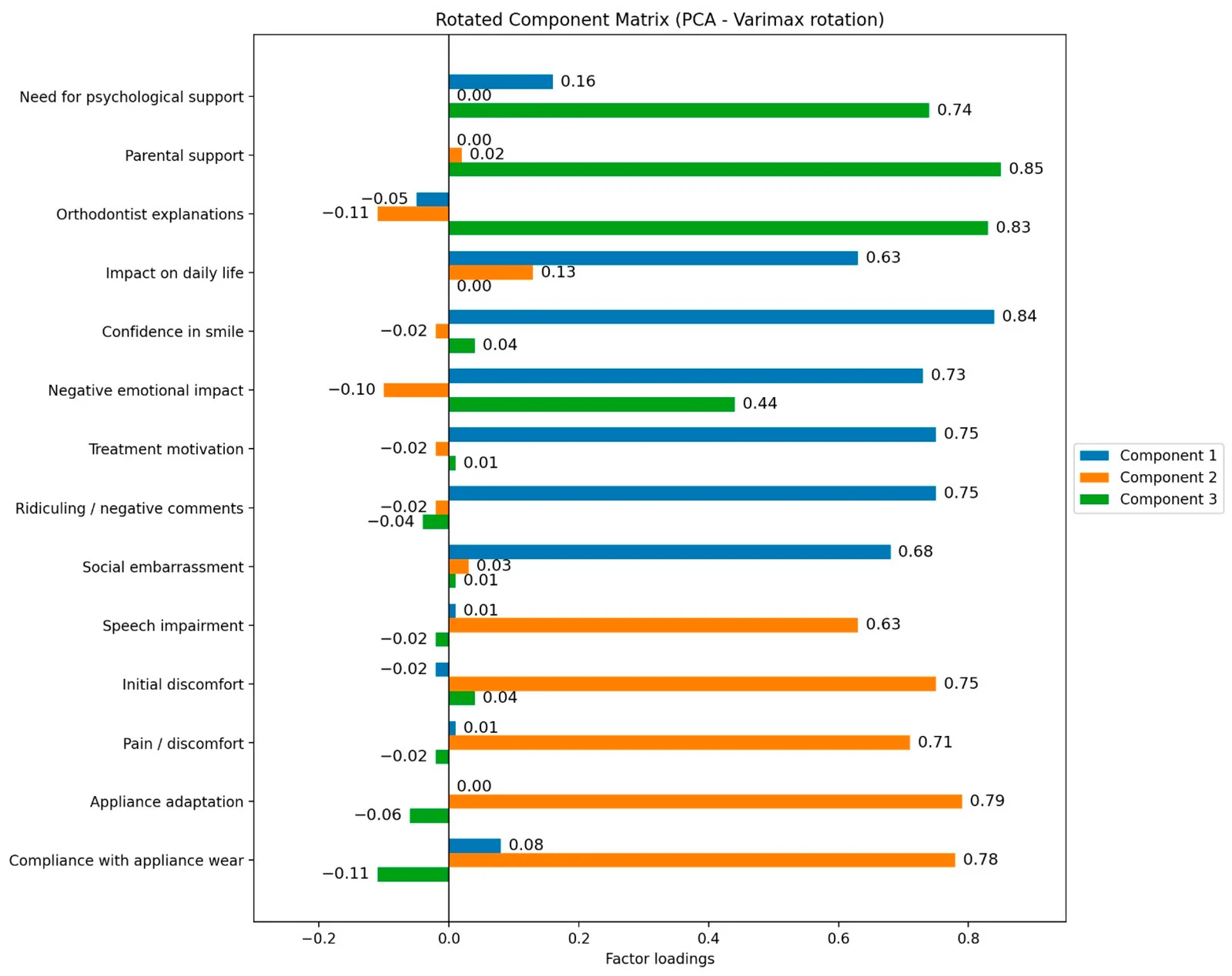
Rotated component matrix obtained by PCA with Varimax rotation.

Varimax rotation with Kaiser normalization produced a clearer three-component solution (Table 15; Figure 3). Component 1 represented the psychological and social perception domain (factor loadings: 0.630–0.844), Component 2 represented compliance and physical discomfort (0.631–0.795), and Component 3 represented the doctor–patient relationship and perceived support (0.745–0.855). Cross-loadings were minimal following rotation, indicating a well-defined factor structure.

## 4. Discussion

The purpose of this methodological study was to report the development and validation stages of a web-based questionnaire designed to evaluate three interrelated domains during orthodontic treatment with removable appliances: compliance, treatment-related discomfort, and psychosocial impact. The present instrument was conceived to support a cross-sectional web-based study and to enable a standardized, patient-centered evaluation of experiences that are often insufficiently captured by clinical indicators alone. This work was structured in accordance with established survey development references and reporting recommendations, including the CHERRIES checklist for web-based surveys [29], as well as broader guidance on quality criteria and methodological rigor in internet survey research [30].

The demographic and treatment-duration profile of the study sample supports its suitability for the present validation aims. The predominance of participants aged 10–13 years is consistent with the high prevalence of interceptive and active orthodontic treatment with removable appliances within this age range, while the modest female predominance (57.6%) mirrors previous reports indicating a higher utilization of orthodontic treatment among female patients. This sex distribution allowed for meaningful comparative analysis of compliance, perception, and psychosocial impact across the sample. Likewise, the finding that 72.0% of participants had been wearing their appliance for at least six months, including 28.0% for more than one year, indicates a high level of treatment persistence within the sample and provided a sufficiently experienced population for a meaningful assessment of compliance, adaptation, and psychosocial impact associated with removable orthodontic treatment.

The psychometric properties observed for the present instrument compare favorably with those reported for other orthodontic and dental PROMs. The internal consistency coefficients obtained across sections (Cronbach’s α ranging from 0.747 to 0.823) fall within the acceptable-to-good range (α = 0.70–0.80) commonly reported for validated instruments assessing oral health-related quality of life and the psychosocial impact of dental treatment, such as the Child Oral Health Impact Profile (COHIP) [1] and the Psychosocial Impact of Dental Aesthetics Questionnaire (PIDAQ) [11,13,31], as well as broader oral health-related quality-of-life measures used in orthodontic populations [2,3,32]. Similarly, the three-factor structure identified through EFA, accounting for 58.69% of the total variance, is consistent with the multidimensional structure typically reported for orthodontic and dental PROMs, which frequently distinguish between functional/physical, psychosocial, and social-support-related domains [33,34,35]. These similarities support the construct validity of the present questionnaire and suggest that it captures dimensions of patient experience comparable to those measured by established instruments, while offering a more treatment-specific focus on removable appliance therapy.

A key rationale for developing a domain-specific instrument is the complexity and variability of patients’ perceptions across treatment phases. Previous research has emphasized that orthodontic patients report nuanced changes in discomfort, daily functioning, and self-perception throughout treatment, indicating that generic measures may not adequately capture orthodontic-specific burdens and adaptations [36,37] Moreover, patient experiences appear multidimensional: compliance behaviors, perceived discomfort, and psychosocial responses are frequently intertwined and can jointly influence satisfaction and outcomes [33,38]. While several validated PROMs exist in dentistry—such as instruments targeting oral health-related quality of life (e.g., OHIP) [2,3,32] or psychosocial effects of dental aesthetics (e.g., PIDAQ) [11,13,31]—these tools are often not designed to simultaneously address the specific combination of wear adherence, appliance-related discomfort, and social/psychological impact that characterizes removable orthodontic therapy. Additionally, the orthodontic questionnaire landscape remains partly fragmented, with instruments frequently targeting narrow endpoints (e.g., satisfaction, pain, aesthetics) rather than providing an integrated view suitable for behavioral and psychosocial interpretation [35,39].

### 4.1. Research Tool

Web-based surveys offer substantial practical advantages for orthodontic research, including rapid dissemination, scalability, cost-efficiency, and convenience for participants, which can facilitate patient-centered data collection in routine clinical contexts and across broader geographic areas [39]. Web-based formats may also increase perceived anonymity, which can encourage more candid reporting of sensitive issues such as embarrassment, distress, or non-adherence [40,41]. These advantages align well with the present instrument’s intention to capture psychosocial impact and adherence-related behaviors with minimal administrative burden.

However, key limitations of web-based surveys must be acknowledged. Selection bias and representativeness concerns may arise because internet access and digital literacy vary across age and socioeconomic strata, potentially excluding or underrepresenting certain patient groups [29,30]. In addition, non-response bias remains a central challenge for online surveys, as participants who drop out or do not respond may differ systematically from completers in motivation, symptom burden, or attitudes [42]. Finally, reliance on self-reported data can introduce recall and social desirability biases, especially for behaviors such as actual wear time and for emotionally loaded constructs [43,44]. These limitations are inherent to the mode of administration and should be considered when interpreting results and generalizing findings.

### 4.2. Questionnaire Format and Length

The relationship between survey length and completion rates remains complex. Many studies suggest that longer web-based questionnaires may reduce completion rates and increase respondent fatigue, potentially encouraging “satisficing” (i.e., providing minimally acceptable rather than fully considered answers) [45,46]. Other work indicates that well-designed surveys can maintain response quality even with a higher number of items, particularly when the survey topic is salient and the structure minimizes burden [47,48]. In the context of orthodontics, relevance and perceived usefulness of the study may further moderate the relationship between length and completion. Therefore, the present survey design should aim to balance comprehensive coverage of constructs against feasibility, using clear language, modular structure, and efficient navigation.

Evidence from broader health questionnaire development suggests that many effective instruments fall within moderate item ranges and that respondent-centered approaches (including feedback from potential respondents) are essential to maintain acceptability [48,49,50]. Accordingly, iterative refinement based on cognitive feedback and pilot testing remains critical to optimize both user experience and data quality.

### 4.3. Conditional Questions (Skip Logic) and Respondent Burden

Conditional branching (skip logic) is widely recognized as a key mechanism for improving relevance and reducing respondent burden in digital surveys. By ensuring that participants only see questions applicable to their experiences, skip logic can reduce unnecessary cognitive load and improve accuracy by preventing responses to irrelevant items [51]. This approach may be especially valuable in orthodontic populations where patient pathways and experiences may differ by age, appliance type, and treatment phase. In addition, structured logic can support quality control by reducing inconsistent response patterns and maintaining a coherent flow [52].

### 4.4. Response Options, Ordering Effects, and Matrix Use

Response option design is a critical determinant of measurement quality. The ordering of response options and the format of scales can introduce response bias (e.g., acquiescence or anchoring), potentially affecting reliability and construct measurement [53]. The literature also indicates that question order and context effects can meaningfully alter self-reported health-related quality-of-life scores, suggesting that survey structure should be carefully considered to minimize systematic bias [54].

Matrix questions, although space-efficient, may increase fatigue and promote straight-lining or satisficing behaviors, particularly when long or repetitive [55,56]. Given mixed evidence regarding their impact on measurement error, matrix use should remain limited and justified, especially in patient populations that may experience discomfort or time constraints. Where matrix formats are used (e.g., Likert-type items for psychosocial impact), careful design and piloting are essential [57].

### 4.5. Content Validity and Expert Review

Content validity is foundational for domain-specific questionnaires and is strengthened through structured expert review. Expert feedback supports relevance, comprehensiveness, and conceptual alignment of items with target constructs—particularly when the instrument aims to integrate behavioral and psychosocial dimensions. This is highly relevant to orthodontics, where psychosocial perceptions may differ across developmental stages and cultural contexts [33,58]. In addition to qualitative input, quantitative content validity indicators (e.g., CVI metrics) are increasingly expected in methodological papers to demonstrate rigorous refinement.

### 4.6. Pilot Testing and Feasibility

Pilot testing provides essential feasibility evidence by evaluating survey flow, item clarity, burden, and preliminary psychometric performance in the target population. The literature indicates that completion rates are influenced by multiple factors beyond length, including topic salience, recruitment strategy, and the perceived value of participation [58]. Therefore, pilot completion rates should be interpreted cautiously and cannot be assumed to replicate in the main study without consistent recruitment conditions.

### 4.7. Limitations

Several limitations must be noted. First, web-based administration inherently increases the risk of selection bias and may limit representativeness, particularly among patients with low digital access or literacy [29,30]. Second, self-reported compliance and experiences are vulnerable to recall and social desirability bias, which may lead to overestimation of adherence or underreporting of psychosocial distress [1]. Third, although content validation and pilot evaluation provide important validity evidence, additional validation is typically required for broader deployment, including construct validation via factor analysis and reliability testing (e.g., internal consistency and test–retest stability) in larger and more diverse samples.

Several additional limitations should also be acknowledged. The cross-sectional design precludes causal or longitudinal inferences regarding how compliance, discomfort, and psychosocial impact evolve across treatment phases or influence clinical outcomes over time. Test–retest reliability was not assessed in the present sample, so the temporal stability of the questionnaire scores remains to be established. Similarly, convergent and discriminant validity were not examined against existing PROMs (e.g., OHIP, COHIP, PIDAQ), which would further strengthen evidence of construct validity [1,11,13,21,35,57]. The factor structure identified here was derived from exploratory factor analysis alone; confirmatory factor analysis in an independent sample is needed to verify its stability and generalizability. Furthermore, the sample was drawn from a single academic center in one country, which may limit the generalizability of the findings to other healthcare settings, populations, and cultural contexts. Finally, questionnaire scores were not correlated with objective clinical indicators of compliance (e.g., wear-time sensors) or treatment outcomes, which would help clarify the clinical validity and predictive utility of the instrument.

### 4.8. Strengths

This study presents several methodological strengths. The questionnaire was developed to reflect the multidimensional nature of orthodontic experience and to address the recognized fragmentation and variability in available orthodontic PROMs [35,59]. By integrating compliance, discomfort, and psychosocial impact within a single framework, the instrument may enable more consistent and comparable assessment of patient-centered outcomes. Additionally, the use of web-based delivery supports scalability and efficient data capture, while iterative refinement—including expert feedback and piloting—strengthens clarity, relevance, and feasibility.

### 4.9. Clinical Implications

The proposed questionnaire may be valuable for both research and clinical applications, particularly for identifying patterns of discomfort and psychosocial burden that could be linked to non-compliance and poorer treatment experience. In routine clinical practice, the instrument could be administered at key treatment checkpoints (e.g., at appliance delivery and at subsequent follow-up visits) to systematically screen for early signs of poor compliance, appliance-related discomfort, or psychosocial distress, allowing clinicians to intervene before these issues compromise treatment outcomes. Patients or families identified through low compliance or high psychosocial-impact scores could be prioritized for targeted counseling, reinforced motivational strategies, or referral for additional psychological support. The instrument may also assist orthodontic teams in tailoring communication strategies to individual patients, for example by clarifying appliance care instructions when scores indicate limited understanding, or by involving parents more directly when perceived family support is low. At a broader level, aggregated questionnaire data could inform quality-improvement initiatives within orthodontic practices or clinics, helping identify systematic gaps in patient education or support that affect multiple patients. Beyond individual patient management, the tool may serve as a standardized outcome measure in comparative effectiveness research, allowing different removable appliance protocols or support interventions to be evaluated from the patient’s perspective rather than relying solely on clinician-assessed outcomes.

### 4.10. Future Research Directions

Future work should expand validation by testing construct validity (e.g., exploratory and confirmatory factor analysis), examining convergent validity against established PROMs (e.g., OHIP, COHIP, PIDAQ), and assessing reliability through internal consistency and test–retest designs [31,34]. Cross-cultural adaptation and validation are also recommended, given the evidence that cultural context can influence satisfaction reporting and psychosocial interpretations in orthodontics [33]. Longitudinal designs that administer the questionnaire at multiple time points across treatment (e.g., at appliance delivery, mid-treatment, and completion) would help clarify how compliance, discomfort, and psychosocial impact evolve over the course of therapy and would allow test–retest reliability to be formally established. Future studies should also examine the predictive validity of the instrument by correlating questionnaire scores with objective clinical indicators, such as sensor-based wear-time monitoring, treatment duration, or achieved clinical outcomes, to determine whether patient-reported scores meaningfully anticipate compliance behavior or treatment success. Multicenter studies involving diverse clinical settings, healthcare systems, and sociodemographic groups would strengthen the generalizability of the psychometric findings beyond the present single-center sample. Finally, confirmatory factor analysis in an independent sample is needed to verify the three-factor structure identified here, and future research could explore whether a shortened version of the instrument retains adequate psychometric properties for use in time-constrained clinical settings. Ultimately, a validated and standardized tool could support patient-centered monitoring, guide targeted educational interventions, and help clinicians identify patients who may require additional psychosocial support to maintain adherence and engagement during treatment.

## 5. Conclusions

The validated questionnaire showed satisfactory reliability and construct validity, with a clear three-factor structure reflecting key dimensions of removable orthodontic treatment. It represents a reliable and practical tool for evaluating compliance, discomfort, and psychosocial impact in children and adolescents undergoing orthodontic therapy.

## Figures and Tables

**Table 1:** Sociodemographic characteristics and duration of use among study participants (n = 250).

Variable	Category	Frequency (n)	Percent (%)	Valid Percent (%)	Cumulative Percent (%)
Age group	Under 10 years	56	22.4	22.4	22.4
	10–13 years	194	77.6	77.6	100.0
	Total	250	100.0	100.0	—
Sex	Female	144	57.6	57.6	57.6
	Male	106	42.4	42.4	100.0
	Total	250	100.0	100.0	—
Duration of use	Under 3 months	31	12.4	12.4	12.4
	3–6 months	57	22.8	22.8	35.2
	6–12 months	92	36.8	36.8	72.0
	Over 1 year	70	28.0	28.0	100.0
	Total	250	100.0	100.0	—

**Table 2:** Descriptive statistics and item–total analysis for Section II—Compliance and physical discomfort (N = 250).

Item	Mean	SD	Corrected Item–Total Correlation	Cronbach’s α If Item Deleted
I wear the removable appliance according to the orthodontist’s recommendation (more than 12 h/day).	3.01	1.01	0.466	0.727
It was easy for me to adapt to wearing the removable appliance.	3.12	1.24	0.459	0.727
Wearing the removable appliance causes pain or physical discomfort.	3.48	1.30	0.293	0.763
The discomfort experienced while wearing the appliance was present only at the beginning of treatment.	3.57	1.11	0.351	0.740
The removable appliance affects my ability to speak normally.	3.80	1.13	0.229	0.779

**Table 3:** Analysis of variance (ANOVA) for items in Section II—Compliance and physical discomfort.

Source	Sum of Squares	df	Mean Square	F	Sig.
Between People	909.987	249	3.655		
Within People—Between Items	107.803	4	26.951	34.708	0.000
Residual	773.397	996	0.777		
Total (Within People)	881.200	1000	0.881		
Total	1791.187	1249	1.434		

Grand Mean = 3.3968.

**Table 4:** Intraclass correlation coefficient (ICC) for Section II—Compliance and physical discomfort.

	Intraclass Correlation	95% Confidence Interval		F Test with True Value 0			
		Lower Bound	Upper Bound	Value	df1	df2	Sig.
Single Measures	0.426	0.366	0.488	4.706	249.0	996	0.000
Average Measures	0.788	0.743	0.826	4.706	249.0	996	0.000

**Table 5:** Descriptive statistics of items in Section III—Psychological and social perception.

Item	Mean	SD	Corrected Item–Total Correlation	Cronbach’s α If Item Deleted
I feel embarrassed to wear the removable appliance in front of colleagues or friends.	2.88	1.30	0.527	0.814
I have been ridiculed or received negative comments because of the removable appliance.	3.28	1.13	0.596	0.793
I feel motivated to continue orthodontic treatment.	2.96	1.11	0.614	0.789
Orthodontic treatment negatively affects my emotional state.	3.19	1.09	0.649	0.782
Wearing the removable appliance increases my confidence in my smile.	3.34	0.94	0.732	0.770
The removable appliance has a significant impact on my daily life.	3.31	0.95	0.465	0.818

**Table 6:** Analysis of variance (ANOVA) for items in Section III—Psychological and social perception.

Source	Sum of Squares	df	Mean Square	F	Sig.
Between People	943.086	249	3.787		
Within People—Between Items	47.139	5	9.428	14.062	0.000
Residual	834.694	1245	0.670		
Total (Within People)	881.833	1250	0.705		
Total	1824.919	1499	1.217		

Grand Mean = 3.1593.

**Table 7:** Intraclass correlation coefficient (ICC) for Section III—Psychological and social perception.

Measure	Intraclass Correlation	95% Confidence Interval	F Test with True Value 0	df1	df2	Sig.
		Lower Bound	Upper Bound	Value		
Single Measures	0.437	0.381	0.496	5.649	249.0	1245
Average Measures	0.823	0.787	0.855	5.649	249.0	1245

**Table 8:** Descriptive statistics of items in Section IV—Doctor–patient relationship and perceived support.

Item	Mean	SD	Corrected Item–Total Correlation	Cronbach’s α If Item Deleted
The orthodontist provided clear explanations regarding how to wear and care for the removable appliance.	3.50	0.78	0.536	0.709
I feel supported by my parents during orthodontic treatment.	3.47	0.68	0.660	0.575
I would benefit from more motivational or psychological support from the orthodontist.	3.36	0.77	0.538	0.707

**Table 9:** Analysis of variance (ANOVA) for items in Section IV—Doctor–patient relationship and perceived support.

Source	Sum of Squares	df	Mean Square	F	Sig.
Between People	276.368	249	1.110		
Within People—Between Items	2.915	2	1.457	5.193	0.006
Residual	139.752	498	0.281		
Total (Within People)	142.667	500	0.285		
Total	419.035	749	0.559		

Grand Mean = 3.4427.

**Table 10:** Intraclass correlation coefficient (ICC) for Section IV—Doctor–patient relationship and perceived support.

Measure	Intraclass Correlation	95% Confidence Interval	F Test with True Value 0	df1	df2	Sig.
		Lower Bound	Upper Bound	Value		
Single Measures	0.496	0.423	0.567	3.955	249.0	498
Average Measures	0.747	0.688	0.797	3.955	249.0	498

**Table 11:** KMO and Bartlett’s test for factor analysis adequacy.

Measure	Value
Kaiser–Meyer–Olkin Measure of Sampling Adequacy	0.713
Bartlett’s Test of Sphericity—Approx. Chi-Square	1303.255
df	91
Sig.	0.000

**Table 12:** Communalities after factor extraction (PCA).

Item	Initial	Extraction
I wear the removable appliance according to the orthodontist’s recommendation (more than 12 h per day).	1.000	0.651
It was easy for me to adapt to wearing the removable appliance.	1.000	0.630
Wearing the removable appliance causes pain or physical discomfort.	1.000	0.507
The discomfort experienced while wearing the appliance was present only at the beginning of treatment.	1.000	0.567
The removable appliance affects my ability to speak normally.	1.000	0.399
I feel embarrassed to wear the removable appliance in front of colleagues or friends.	1.000	0.456
I have been ridiculed or received negative comments because of the removable appliance.	1.000	0.561
I feel motivated to continue orthodontic treatment.	1.000	0.567
Orthodontic treatment negatively affects my emotional state.	1.000	0.735
Wearing the removable appliance increases my confidence in my smile.	1.000	0.714
The removable appliance has a significant impact on my daily life.	1.000	0.413
The orthodontist provided clear explanations regarding how to wear and care for the removable appliance.	1.000	0.705
I feel supported by my parents during orthodontic treatment.	1.000	0.732
I would benefit from more motivational or psychological support from the orthodontist.	1.000	0.580

**Table 13:** Total variance explained by principal components (PCA).

Component	Initial Eigenvalues			Extraction Sums of Squared Loadings			Rotation Sums of Squared Loadings		
	Total	% of Variance	Cumulative %	Total	% of Variance	Cumulative %	Total	% of Variance	Cumulative %
1	3.378	24.127	24.127	3.378	24.127	24.127	3.255	23.248	23.248
2	2.831	20.221	44.348	2.831	20.221	44.348	2.770	19.786	43.034
3	2.008	14.340	58.688	2.008	14.340	58.688	2.192	15.654	58.688

**Table 14:** Unrotated component matrix (Component Matrix) obtained by principal component analysis (PCA).

Item	Component 1	Component 2	Component 3
I wear the removable appliance according to the orthodontist’s recommendation (more than 12 h per day).	−0.006	0.801	0.099
It was easy for me to adapt to wearing the removable appliance.	−0.073	0.774	0.162
Wearing the removable appliance causes pain or physical discomfort.	−0.062	0.675	0.217
The discomfort experienced while wearing the appliance was present only at the beginning of treatment.	−0.054	0.707	0.253
The removable appliance affects my ability to speak normally.	−0.035	0.611	0.153
I feel embarrassed to wear the removable appliance in front of colleagues or friends.	0.639	0.130	−0.175
I have been ridiculed or received negative comments because of the removable appliance.	0.695	0.088	−0.263
I feel motivated to continue orthodontic treatment.	0.716	0.094	−0.213
Orthodontic treatment negatively affects my emotional state.	0.834	−0.102	0.172
Wearing the removable appliance increases my confidence in my smile.	0.813	0.098	−0.210
The removable appliance has a significant impact on my daily life.	0.587	0.218	−0.144
The orthodontist provided clear explanations regarding how to wear and care for the removable appliance.	0.193	−0.318	0.753
I feel supported by my parents during orthodontic treatment.	0.271	−0.188	0.790
I would benefit from more motivational or psychological support from the orthodontist.	0.386	−0.157	0.638

**Table 15:** Rotated component matrix (Rotated Component Matrix) obtained by PCA with Varimax rotation.

Item	Component 1	Component 2	Component 3
I wear the removable appliance according to the orthodontist’s recommendation (more than 12 h per day).	0.084	0.795	−0.106
It was easy for me to adapt to wearing the removable appliance.	−0.001	0.791	−0.063
Wearing the removable appliance causes pain or physical discomfort.	−0.020	0.711	0.015
The discomfort experienced while wearing the appliance was present only at the beginning of treatment.	−0.019	0.751	0.043
The removable appliance affects my ability to speak normally.	0.013	0.631	−0.020
I feel embarrassed to wear the removable appliance in front of colleagues or friends.	0.675	0.034	0.008
I have been ridiculed or received negative comments because of the removable appliance.	0.747	−0.035	−0.045
I feel motivated to continue orthodontic treatment.	0.753	−0.016	0.006
Orthodontic treatment negatively affects my emotional state.	0.726	−0.105	0.444
Wearing the removable appliance increases my confidence in my smile.	0.844	−0.018	0.038
The removable appliance has a significant impact on my daily life.	0.630	0.131	−0.001
The orthodontist provided clear explanations regarding how to wear and care for the removable appliance.	−0.079	−0.108	0.829
I feel supported by my parents during orthodontic treatment.	0.004	0.020	0.855
I would benefit from more motivational or psychological support from the orthodontist.	0.160	0.001	0.745

## Data Availability

The original contributions presented in this study are included in the article and supplementary material. Further inquiries can be directed to the corresponding author.

## Supplementary Materials

The following supporting information can be downloaded at https://www.mdpi.com/article/10.3390/dj14090543/s1, Supplementary File S1: Questionnaire: Experience of Using a Removable Orthodontic Appliance.

## Abbreviations

The following abbreviations are used in this manuscript:

ANOVA	Analysis of Variance
ICC	Intraclass Correlation Coefficient
KMO	Kaiser–Meyer–Olkin Measure of Sampling Adequacy
KAP	Knowledge, Attitudes, and Practices
PCA	Principal Component Analysis
SD	Standard Deviation
CI	Confidence Interval
MS	Mean Square
CHERRIES	Checklist for Reporting Results of Internet E-Surveys
PROMs	Patient-Reported Outcome Measures
OHIP	Oral Health Impact Profile
PIDAQ	Psychosocial Impact of Dental Aesthetics Questionnaire
CVI	Content Validity Index
COHIP	Child Oral Health Impact Profile

## References

[1] Dunlow N., Phillips C., Broder H.L. (2007). Concurrent validity of the COHIP. Community Dent. Oral Epidemiol..

[2] Kavaliauskienė A., Šidlauskas A., Zaborskis A. (2017). Association between Global Life Satisfaction and Self-Rated Oral Health Conditions among Adolescents in Lithuania. Int. J. Environ. Res. Public Health.

[3] Matilainen L.B., Johansson K., Petrén S., Brechter A., Wiaderny M., Michelotti A., Paulsson L. (2026). Oral health-related quality of life, experience and satisfaction in adolescents treated for dental crowding with self-ligating or conventional fixed appliances: A multicentre randomized controlled trial. Eur. J. Orthod..

[4] Romero-Maroto M., Santos-Puerta N., González-Olmo M.J. (2015). The impact of dental appearance and anxiety on self-esteem in adult orthodontic patients. Orthod. Craniofacial Res..

[5] Santamaría-Villegas A., Manrique R., Álvarez-Varela E., Restrepo-Serna C. (2017). Effect of removable functional appliances on mandibular length in patients with class II with retrognathism: Systematic review and meta-analysis. BMC Oral Health.

[6] Singaraju G.S., Obili S., Nettam V., Vatturu S., Erugu S. (2021). Impact of self-esteem on the relationship between orthodontic treatment and the oral health-related quality of life in patients after orthodontic treatment—A systematic review. Med. Pharm. Rep..

[7] Talpoş Ş., Pricop M., Szuhanek C., Avramut R., Nikolajević-Stoican N., Maracineanu R., Talpos R., Hajaj T., Popa M. (2023). Age-Related Quality of Life and Psychosocial Impact of Chin Asymmetry in Adolescents and Young Adults Undergoing Orthodontic and Orthognathic Correction. Healthcare.

[8] Bradley E., Shelton A., Hodge T., Morris D., Bekker H., Fletcher S., Barber S. (2020). Patient-reported experience and outcomes from orthodontic treatment. J. Orthod..

[9] Aprile M., Verdecchia A., Dettori C., Spinas E. (2025). Malocclusion and its relationship with sound speech disorders in deciduous and mixed dentition: A scoping review. Dent. J..

[10] Gilchrist F., Marshman Z. (2020). Patient-reported Outcomes (PROs) in clinical trials in paediatric dentistry. Int. J. Paediatr. Dent..

[11] González H., Ortuño D., Macherone C., Zedan Y., Torres M. (2023). Cross-cultural adaptation and validation of the PIDAQ questionnaire in Chilean adolescents with oral malocclusion. Res. Sq..

[12] Khalil R., Yaqoob H., Ilyas M.R., Sukhia R.H., Mubassar F. (2026). Association between Clark twin block appliance treatment and social and emotional wellness in children with malocclusion—A cohort study. BMC Psychol..

[13] Oneto H.G., Pinto M.I.T., Abuawad Y.Z., Chaparro M.M., Buratovic J.P.V., Ortuño D. (2024). Cross-cultural adaptation of PIDAQ questionnaire to evaluate the psychosocial impact of dental esthetics in Chilean adolescents with malocclusion. Res. Sq..

[14] Santiwong P., Sommaluan K., Mokkasak S., Rachuratchata C., Rattanaopas T., Sipiyaruk K. (2022). The Implementation of PROMs/PREMs in the Assessment of Orthodontic Treatment Outcomes. J. Int. Soc. Prev. Community Dent..

[15] Tassi A., Tan J., Piplani B., Longmire N.M., Riff K.W.Y.W., Klassen A.F. (2021). Establishing content validity of an orthodontic subset of the FACE-Q Craniofacial Module in children and young adults with malocclusion. Orthod. Craniofacial Res..

[16] Vale F., Travassos R., Couto I.S.L., Ribeiro M., Marques F., Caramelo F., Marto C.M., Spagnuolo G., Paula A., Nunes C.S. (2024). Patient’s Perspective on Miniscrews During Orthodontic Treatment—A Systematic Review with Meta-Analysis. Orthod. Craniofacial Res..

[17] Zhao J., Feng Z., Liu Y., Sun S., Feng Z. (2025). Advances in orthodontic treatment for periodontal disease: A bibliometric analysis, emerging insights and clinical implications. Front. Dent. Med..

[18] Krishnakumar K., Kalaskar R. (2023). Evaluation of Children’s Attitude toward Conventional Removable Appliances and Novel Vivid Pedo Appliances: A Randomized Controlled Trial. Int. J. Clin. Pediatr. Dent..

[19] Elkhatib A.A., Gad A. (2024). Patient and Parental Satisfaction Following Treatment of Anterior Crossbite in Mixed Dentition: A Comparative Study of Cemented Lower Inclined Plane (CLIP) and Removable Anterior Expansion Screw (RAES). https://assets-eu.researchsquare.com/files/rs-4227859/v1/a5bfdaf0-a6a5-4c61-a8fe-9ece1ae8f72c.pdf.

[20] Kalaoğlu E.E., Gök G.D. (2024). Comparison of acceptability of orthodontic appliances in children in mixed dentition treated with removable acrylic appliances and Invisalign first: A cross-sectional study. BMC Oral Health.

[21] Gatti-Reis L., Alvarenga R.N., Ju X., Jamieson L., Abreu L.G., Paiva S.M. (2025). Psychometric properties of the Brazilian version of the Patient Satisfaction Questionnaire. Braz. Dent. J..

[22] Martina Z., Vedrana T., Palomo J.M., Špalj S. (2025). Mandibular Advancement in Dentofacial Orthopaedics: Effects on Pharyngeal Airway, Dentoskeletal Characteristics and Quality of Life: A Randomised Controlled Trial. Orthod. Craniofacial Res..

[23] Tang B., Ting J., Brown R., Nathan S., Ashton-James C.E., Sadr A., Gholamrezaei A. (2023). Is there an association between clinician behavioural factors, and the experience of pain in a dental setting?. A Scoping Rev..

[24] Rai K., Kote A.R., Priya K., Shreya S., Saha S. (2025). Effectiveness of Clear Aligner Therapy in Orthodontic Management for Children with Mixed Dentition: A Scoping Review. Int. J. Clin. Pediatr. Dent..

[25] Lorek M., Jarząbek A., Sycińska-Dziarnowska M., Gołąb S., Spagnuolo G., Woźniak K., Szyszka-Sommerfeld L. (2024). The association between patients’ personality traits and pain perception during orthodontic treatment: A systematic review. Front. Neurol..

[26] AboulAzm N., Sharaf A., Dowidar K., Abdelhakim A. (2023). effectiveness of CAD/CAM peek fabricated space maintainer in children: A randomized controlled clinical trial. Alex. Dent. J..

[27] Koaban A., Alharbi S., Al-Shehri A.Z., Al-Shamri B.S., Aburazizah M.F., Al-Qahtani G.H., Al-Wusaybie L.H., Alkhalifa L.B., Al-Saad M.M., Al-Nehab A.A. (2024). Current Trends in Pediatric Orthodontics: A Comprehensive Review. Cureus.

[28] Damanhouri W.H., Moussa K., Bathallath J., Alsomali Z., Bakor A., Attar M.H. (2025). Management of a pediatric patient with dental anomalies and its effect on psychosocial status: A case report. Front. Dent. Med..

[29] Eysenbach G., Wyatt J. (2002). Using the Internet for surveys and health research. J. Med. Internet Res..

[30] Braekman E., Charafeddine R., Demarest S., Drieskens S., Tafforeau J. (2022). Comparing web-based versus face-to-face and paper-and-pencil surveys: Effects on response quality and representativeness. Soc. Sci. Comput. Rev..

[31] Alharbi R., Eshky R., Marae S., Hifnawy T., Alsulaimani M. (2020). Translation and validation of the Arabic version of the Psychosocial Impact of Dental Aesthetics Questionnaire (PIDAQ). J. Orthod. Sci..

[32] Jaber S.T., Hajeer M.Y., Burhan A.S., Latifeh Y. (2022). The effect of treatment with clear aligners versus fixed appliances on oral health-related quality of life in patients with severe crowding: A one-year follow-up randomized controlled clinical trial. Cureus.

[33] Yovanka V., Kusnoto J., Andayani L. (2023). Characteristics of orthodontic appliance users based on demographics, self-perception, psychosocial, and oral disorders (study among undergraduate students in West Jakarta). J. Indones. Dent. Assoc..

[34] Oh M., Kim E., Park A., Kim M., Cho J. (2020). Part I. What drives Korean adults to seek orthodontic treatment: Reliability and validity of a measurement instrument for the perception of orthodontic treatment. Korean J. Orthod..

[35] Tidbury K., Sayers M., Andiappan M., Newton T. (2021). Psychometric validation of a pre-existing questionnaire used to measure patient satisfaction following orthodontic treatment in a UK population. J. Orthod..

[36] Wiedel A.P., Bondemark L. (2016). A randomized controlled trial of self-perceived pain, discomfort, and impairment of jaw function in children undergoing orthodontic treatment with fixed or removable appliances. Angle Orthod..

[37] Alansari R. (2024). Orthodontic patient satisfaction: Validation of an Arabic patient satisfaction questionnaire. APOS Trends Orthod..

[38] Harlachova T., Tserakhava T. (2022). Dental aspects of quality of life in orthodontic patients. Pediatr. Dent. Dent. Profilaxis.

[39] Howell R.T., Rodzon K.S., Kurai M., Sanchez A.H. (2010). A validation of well-being and happiness surveys for administration via the Internet. Behav. Res. Methods.

[40] Wallin E.E.K., Mattsson S., Olsson E.M.G. (2016). The preference for Internet-based psychological interventions by individuals without past or current use of mental health treatment delivered online: A survey study with mixed-methods analysis. JMIR Ment. Health.

[41] Muhuri P.K. (2022). Assessing the potential for nonresponse bias and measurement concordance in the clinical preventive services self-administered questionnaire survey. J. Econ. Soc. Meas..

[42] Jones M., Calzavara L., Allman D., Worthington C., Tyndall M., Iveniuk J. (2016). A comparison of web and telephone responses from a national HIV and AIDS survey. JMIR Public Health Surveill..

[43] Milton A.C., Ellis L.A., Davenport T.A., Burns J.M., Hickie I.B. (2017). Comparison of self-reported telephone interviewing and web-based survey responses: Findings from the second Australian Young and Well National Survey. JMIR Ment. Health.

[44] Mavletova A. (2013). Data quality in PC and mobile web surveys. Soc. Sci. Comput. Rev..

[45] Mühlböck M., Steiber N., Kittel B. (2017). Less supervision, more satisficing? Comparing completely self-administered web-surveys and interviews under controlled conditions. Stat. Politics Policy.

[46] Dykema J., Jones N., Piché T., Stevenson J. (2013). Surveying clinicians by web. Eval. Health Prof..

[47] Gregory M.E., Huerta T.R., McAlearney A.S. (2023). Implications for electronic surveys in inpatient settings based on patient survey response patterns: Cross-sectional study. J. Med. Internet Res..

[48] Hsu C., Ståhl D., Mouchlianitis E., Peters E., Vamvakas G., Keppens J., Yiend J. (2023). User-centered development of STOP (Successful Treatment for Paranoia): Material development and usability testing for a digital therapeutic for paranoia. JMIR Hum. Factors.

[49] Pfennings L.E.M., van der Ploeg H.M., Cohen L., Bramsen I., Polman C.H., Lankhorst G.J., Vleugels L. (1999). A health-related quality of life questionnaire for multiple sclerosis patients. Acta Neurol. Scand..

[50] Ahmed R., Robinson R., Elsony A., Thomson R., Squire S.B., Malmborg R., Mortimer K. (2018). A comparison of smartphone and paper data-collection tools in the Burden of Obstructive Lung Disease (BOLD) study in Gezira state, Sudan. PLoS ONE.

[51] Yang D., Toth D. (2022). Analyzing the association of objective burden measures to perceived burden with regression trees. J. Off. Stat..

[52] Lelkes Y., Weiss R. (2015). Much ado about acquiescence: The relative validity and reliability of construct-specific and agree–disagree questions. Res. Polit..

[53] Tempel T., Schneider L. (2024). A good life with spina bifida: Experimental evidence on how question order influences outcomes when asking about quality of life. Discov. Psychol..

[54] Chyung S.Y., Kennedy M., Campbell I. (2018). Evidence-based survey design: The use of ascending or descending order of Likert-type response options. Perform. Improv..

[55] Knaüper B., Carrière K., Chamandy M., Xu Z., Schwarz N., Rosen N. (2016). How aging affects self-reports. Eur. J. Ageing.

[56] Zhang X., Tse W.S., Savalei V. (2019). Improved properties of the Big Five Inventory and the Rosenberg Self-Esteem Scale in the expanded format relative to the Likert format. Front. Psychol..

[57] Haik A., Yassir Y.A. (2024). Development of a questionnaire for patient perception to functional appliances. BMC Oral Health.

[58] Rodrigues B., Encantado J., Carraça E., Martins J., Marques A., Lopes L., Santos R. (2023). Questionnaires measuring 24-hour movement behaviors in childhood and adolescence: Content description and measurement properties—A systematic review. J. Phys. Act. Health.

[59] Fricker R.D., Schonlau M. (2002). Advantages and disadvantages of Internet research surveys: Evidence from the literature. Field Methods.

