# Supplementary material for: Development and Validation of a Patient-Reported Outcome Questionnaire for Children Undergoing Removable Orthodontic Treatment
Source: Dent J (Basel). 2026 Sep 1;14(9):543. doi: 10.3390/dj14090543 (PMC13605303; doi:10.3390/dj14090543)
Supplement: Supplementary file 1 [file dentistry-14-00543-s001.zip › dentistry-4470860-supplementary.pdf]

Questionnaire: Experience of Using a Removable Orthodontic Appliance

You are invited to participate in this study, which aims to evaluate the experience of patients wearing a removable orthodontic appliance. We seek to better understand the difficulties encountered, the level of comfort, and perceptions related to wearing this type of appliance, as well as its impact on daily life.

By completing this anonymous questionnaire, you will help us identify aspects that can be improved in the dentist–patient relationship and in optimizing orthodontic treatment.

Completing the questionnaire takes approximately 5 minutes. Your responses will be treated confidentially and will be used exclusively for academic purposes, within the framework of a scientific article.

Thank you for your time and for your valuable contribution to this research!

Section I – General Information

1. **Age:**
   ☐ Under 10 years
   ☐ 10–13 years
2. **Sex:**
   ☐ Female
   ☐ Male
3. **How long have you been wearing the removable appliance?**
   ☐ Less than 3 months
   ☐ 3–6 months
   ☐ 6–12 months
   ☐ More than 1 year

Section II – Compliance and Physical Discomfort

4. **On average, how many hours per day do you wear the appliance?**
   ☐ Less than 4 hours
   ☐ 4–8 hours
   ☐ 8–12 hours
   ☐ According to the orthodontist’s recommendation (more than 12 hours)
5. **Was it easy for you to get used to wearing the appliance?**
   ☐ Yes

☐ No
☐ Partially

6. **Did you experience pain or discomfort while wearing the appliance?**
   ☐ Yes, frequently
   ☐ Only at the beginning
   ☐ Very rarely
   ☐ Never

7. **Does the appliance affect your speech?**
   ☐ Yes, significantly
   ☐ Slightly
   ☐ No

Section III – Psychological and Social Perception

8. **Do you feel embarrassed wearing the appliance in front of classmates/friends?**
   ☐ Yes
   ☐ Sometimes
   ☐ No

9. **Have you ever been teased or commented on because of the appliance?**
   ☐ Yes
   ☐ No

10. **How do you feel about your orthodontic treatment?**
    ☐ Very motivated
    ☐ Indifferent
    ☐ I would like to quit, but I continue
    ☐ I regret starting the treatment

11. **Do you think the removable appliance influences your daily life?**
    ☐ Yes, negatively
    ☐ Yes, positively (I feel more confident about my smile)
    ☐ It has no noticeable impact

Section IV – Relationship with the Orthodontist and Provided Support

12. **Did you receive clear explanations from your orthodontist regarding how to wear and care for the appliance?**
    ☐ Yes

☐ No
☐ Partially

13. **Do you feel supported by your parents during the treatment?**
☐ Yes
☐ No
☐ Sometimes

14. **Would you like your orthodontist to provide more motivational or psychological advice?**
☐ Yes
☐ No
☐ I do not know
